# Systolic Compression of Intramural Coronary Arteries in Hypertrophic Cardiomyopathy

**DOI:** 10.1155/2012/629842

**Published:** 2012-01-18

**Authors:** Muhammet Rasit Sayin, Sait Mesut Dogan, Turgut Karabag, Ibrahim Akpinar, Mustafa Aydin

**Affiliations:** Department of Cardiology, School of Medicine, Zonguldak Karaelmas University, Kozlu, Zonguldak 67600, Turkey

## Abstract

We report a case of hypertrophic cardiomyopathy due to systolic total narrowing of side branches of all major coronary arteries.

## 1. Introduction

 Hypertrophic cardiomyopathy (HCM) is characterized by unexplained left ventricular hypertrophy (LVH) that develops in the absence of predisposing cardiac conditions (e.g., aortic stenosis) or cardiovascular conditions (e.g., hypertension) [1]. The clinical manifestations of HCM range from asymptomatic to progressive heart failure and vary between individuals even within the same family [1]. HCM is often associated with disabling symptoms, arrhythmias, and sudden cardiac death [2]. Myocardial ischemia is thought to be important contributors for sudden death [2]. Several mechanisms are potentially responsible for ischemia in HCM, including the obstruction of epicardial and intramyocardial arteries by systolic compression [3, 4]. In this paper, we report a case of HCM due to systolic total narrowing of side branches of all major coronary arteries.

## 2. Case Report

 A 43-year-old woman was admitted to the emergency department with a presyncope episode of fifteen minutes duration. She also had had angina pectoris, palpitation, and shortness of breath on exertion for 6 months. The patient has a history of known HCM with a medication of metoprolol succinate 25 mg twice a day. She did not have any cardiovascular risk factors except smoking. Vital signs showed a blood pressure of 120/80 mmHg and a heart rate of 52 beats/minute. Physical examination was entirely normal except 2/6 systolic murmur at apex. The electrocardiogram (ECG) at admission showed a prolongation of *P* waves and voltage criteria of LVH. The posteroanterior chest X-ray showed enhanced cardiothoracic indexes. There was no arrhythmic episode on telemetric ECG recordings.

 Echocardiography revealed an ejection fraction of 65%, and increased interventricular septum to posterior wall thickness ratio as 2.25. Enlarged left atrium (5.1 cm), enlarged right atrium (4.1 cm), mild mitral insufficiency, and moderate tricuspid insufficiency were also seen. Gradient on left ventricular outflow track and systolic anterior motion were not detected.

**Figure 1:**
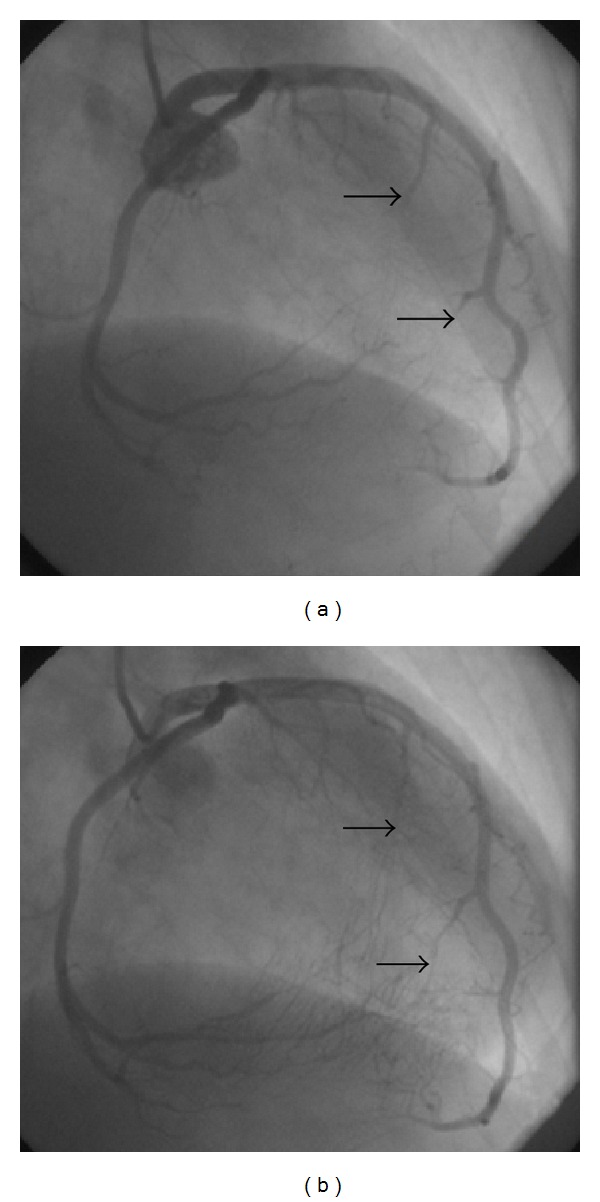
Systolic compression of septal perforators of LAD (a) is seen during systole whereas arteries are fully opened during diastole (b).

**Figure 2:**
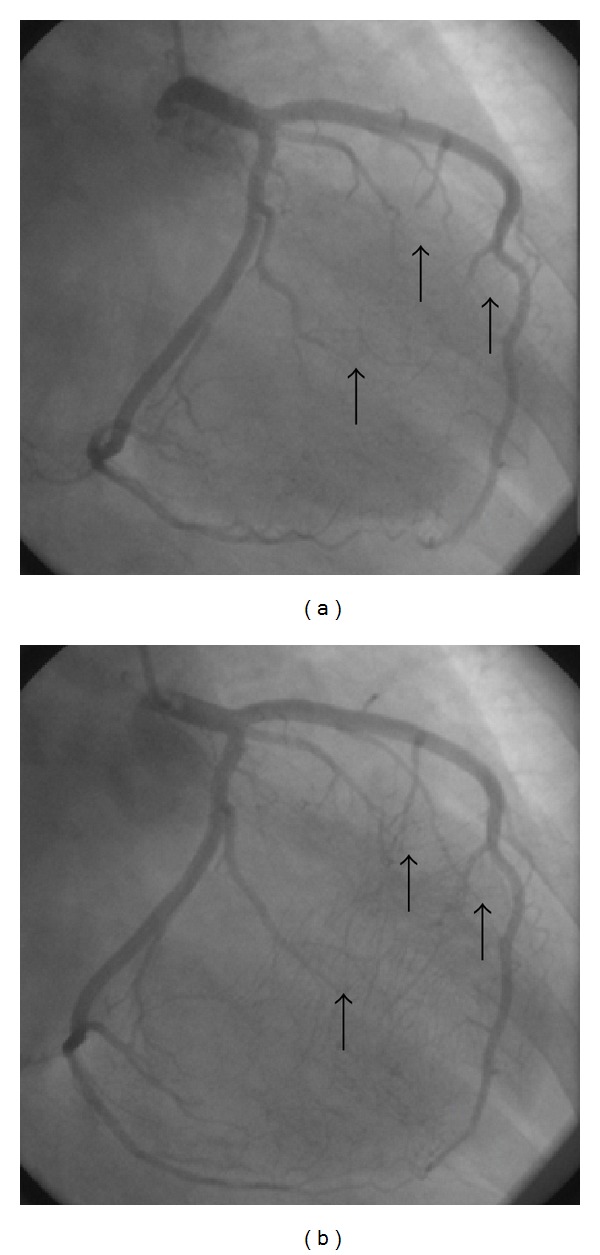
Systolic compression of marginal branches of LCx (a) is seen during systole whereas arteries are fully opened during diastole (b).

**Figure 3:**
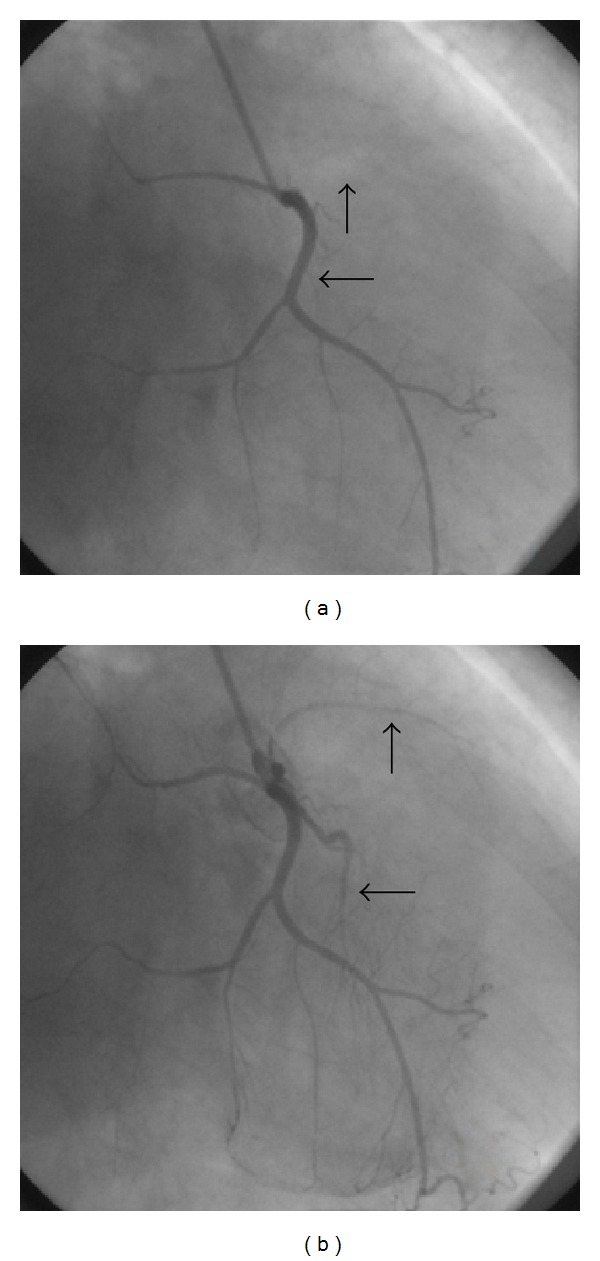
Systolic compression of side branches of RCA (a) is seen during systole whereas arteries are fully opened during diastole (b).

 Because of typical chest pain, coronary angiography was performed. Coronary angiography revealed total occlusion and flow interruption in the intramural parts of the coronary arteries during systole and disappearance with diastole (Figures 1, 2, and 3). No visible atherosclerotic plaque was seen.

## 3. Discussion

 HCM is a primary hypertrophy of cardiac muscle associated with a small left ventricular cavity, increased systolic function, and impaired diastolic function. The clinical diagnosis of HCM is established more easily and reliably with two-dimensional echocardiography by demonstrating LVH (typically asymmetric in distribution and showing virtually any diffuse or segmental pattern of left ventricular wall thickening) [5, 6].

 Systolic compression of the epicardial coronary arteries inside overlying myocardial tissue is known as myocardial bridge (MB). MB has been traditionally viewed as a benign condition with favorable long-term prognosis. MB is generally confined to the mid-left anterior descending artery (LAD) [7] and the main angiographic finding is systolic compression of the involved epicardial coronary artery [8]. Prolonged pressure on coronary arteries from myocardium during systole and early diastole may hinder coronary blood flow and lead to severe ischemic events.

 HCM is often associated with disabling symptoms, arrhythmias, and sudden cardiac death [2]. Myocardial ischemia is thought to be important contributors to sudden cardiac death [2]. Several mechanisms are potentially responsible for ischemia in HCM. Myocardial perfusion may also be limited by elevated left ventricular diastolic pressure, abbreviated diastolic intervals, and systolic arterial compression [9]. Coronary flow reserve limitation in HCM is often most striking in the subendocardium and does not typically show single coronary artery distribution [10].

 Coronary compression is a more common mechanism in HCM and myocardial hyperdynamic and hypertrophic adaptations may extend to previously silent bridges that become compressive. Coronary compression is found in 30% to 80% of adults who have HCM [11, 12]. In HCM, angiographic obliteration in systole of the septal perforator branches of the LAD and posterior descending artery (often called septal blanching) occurs commonly [13]. In our case angiographic obliteration was seen not only septal perforators of LAD artery, but also marginal branch of Cx artery and sinus node artery of RCA which are thought to cause of ischemia. As far as we know, this is the first case reporting the systolic compression of the side branches of all three coronary arteries.

 Therapeutic approaches that have been attempted for myocardial bridging include beta blockers, calcium channel blockers, stents, minimally invasive coronary artery bypass grafting, and surgical myotomy [6]. In HCM, compression of the coronary arteries may be of additional importance in limiting coronary flow.

## References

[1] Cirino AL, Ho C, Pagon RA, Bird TC, Dolan CR, Stephens K (1993–2008). Familial hypertrophic cardiomyopathy overview. *GeneReviews*.

[2] McKenna WJ, Behr ER (2002). Hypertrophic cardiomyopathy: management, risk stratification, and prevention of sudden death. *Heart*.

[3] Cannon RO, Baroldi G, Camerini F, Goodwin JF (1990). Ischemia, coronary blood flow and coronary reserve in hypertrophic cardiomyopathy. *Advances in Cardiomyopathy*.

[4] Schwartzkopff B, Mundhenke M, Strauer BE (1998). Alterations of the architecture of subendocardial arterioles in patients with hypertrophic cardiomyopathy and impaired coronary vasodilator reserve: a possible cause for myocardial ischemia. *Journal of the American College of Cardiology*.

[5] Klues HG, Schiffers A, Maron BJ (1995). Phenotypic spectrum and patterns of left ventricular hypertrophy in hypertrophic cardiomyopathy: morphologic observations and significance as assessed by two-dimensional echocardiography in 600 patients. *Journal of the American College of Cardiology*.

[6] Maron BJ, McKenna WJ, Danielson GK (2003). American College of Cardiology/European Society of Cardiology Clinical Expert Consensus Document on Hypertrophic Cardiomyopathy: a report of the American College of Cardiology Foundation Task Force on Clinical Expert Consensus Documents and the European Society of Cardiology Committee for Practice Guidelines. *European Heart Journal*.

[7] Irvin RG (1982). The angiographic prevalence of myocardial bridging in man. *Chest*.

[8] Portmann WC, Iwig J (1960). Die intramurale koronarie im angiogramm. *Fortschr Geb Rontgenstr Nuklearmed*.

[9] Cannon RO, Rosing DR, Maron BJ (1985). Myocardial ischemia in patients with hypertrophic cardiomyopathy: contribution of inadequate vasodilator reserve and elevated left ventricular filling pressures. *Circulation*.

[10] Cannon RO, Dilsizian V, O'Gara PT (1991). Myocardial metabolic, hemodynamic, and electrocardiographic significance of reversible Thallium-201 abnormalities in hypertrophic cardiomyopathy. *Circulation*.

[11] Angelini P, Trivellato M, Donis J, Leachman RD (1983). Myocardial bridges: a review. *Progress in Cardiovascular Diseases*.

[12] Kitazume H, Kramer JR, Krauthamer D, El Tobgi S, Proudfit WL, Sones FM (1983). Myocardial bridges in obstructive hypertrophic cardiomyopathy. *American Heart Journal*.

[13] Mohiddin SA, Fananapazir L (2002). Systolic compression of epicardial coronary and intramural arteries: in children with hypertrophic cardiomyopathy. *Texas Heart Institute Journal*.

